# The Reflective Mind of the Anxious in Action: Metacognitive Beliefs and Maladaptive Emotional Regulation Strategies Constrain Working Memory Efficiency

**DOI:** 10.3390/ejihpe14030034

**Published:** 2024-02-26

**Authors:** François-Xavier Cécillon, Martial Mermillod, Christophe Leys, Hippolyte Bastin, Jean-Philippe Lachaux, Rebecca Shankland

**Affiliations:** 1 Laboratoire Développement Individu Processus Handicap Education, Université Lumière Lyon 2, 5, Avenue Pierre Mendès-France, 69676 Bron, CEDEX, France; hippolyte.bastin@psycho-prat.fr (H.B.); rebecca.shankland@univ-lyon2.fr (R.S.); 2 Laboratoire Psychologie et NeuroCognition, CNRS, Université Grenoble Alpes, 38000 Grenoble, France; 3 Faculté de Psychologie, Sciences de l’Education et Logopédie, Université Libre de Bruxelles, Avenue Franklin Roosevelt, 50-CP191, 1050 Bruxelles, Belgium; christophe.leys@ulb.be; 4 Centre de Recherche en Neurosciences de Lyon, Bâtiment 452—95 Bd Pinel, 69500 Bron, France; jp.lachaux@inserm.fr; 5 Institut Universitaire de France, 1 Rue Descartes, 75231 Paris, CEDEX 05, France

**Keywords:** attentional control theory, executive function, anxiety, emotion regulation, metacognitive belief, working memory

## Abstract

The Attentional Control Theory (ACT) posits that, while trait anxiety may not directly impact performance, it can influence processing efficiency by prompting the use of compensatory mechanisms. The specific nature of these mechanisms, which might be reflective, is not detailed by the ACT. In a study involving 110 students (M = 20.12; SD = 2.10), surveys were administered to assess the students’ metacognitive beliefs, trait anxiety, and emotion regulation strategies (ERSs). The participants engaged in two working memory exercises: the digit span task from the WAIS-IV and an emotional n-back task. The findings indicated that anxiety, metacognitive beliefs, and maladaptive ERSs did not affect task performance but were correlated with increased response times. Several regression analyses demonstrated that a lack of confidence in one’s cognitive abilities and maladaptive ERSs predict higher reaction times (RT) in the n-back task. Additionally, maladaptive ERSs also predict an increased use of strategies in the digit span task. Finally, two mediation analyses revealed that anxiety increases processing efficiency, and this relation is mediated by the use of maladaptive ERSs. These results underscore the importance of the reflective level in mediating the effects of trait anxiety on efficiency. They highlight the necessity of incorporating metacognitive beliefs and maladaptive emotion regulation strategies for a thorough comprehension of the Attentional Control Theory. Recognizing these factors offers valuable perspectives for enhancing cognitive capabilities and fostering academic achievement.

## 1. Introduction

When faced with a stressful situation, such as an exam, we do not react in the same way in attempting to manage our anxiety and optimize our abilities for success. Our experiences are specific to the situation but also to our predisposition to experiencing anxiety [1,2]. These anxious traits are characterized by worry, intrusive thoughts, physiological manifestations, and difficulty detaching from negative stimuli [2]. They would hinder the mobilization of a cognitive function that is particularly essential for learning and academic achievement: Working Memory (WM) [3]. Indeed, one of the most comprehensive definitions of WM is as follows: “The ensemble of components of the mind that hold a limited amount of information temporarily in a heightened state of availability for use in ongoing information processing” [4]. The primary focus of this article will be to examine how trait anxiety impacts working memory and to explore the key factors that contribute to its maintenance and enhancement.

### 1.1. Trait Anxiety and Working Memory

The connection between trait anxiety and working memory (WM) is complex, with initial research producing varied and sometimes contradictory outcomes. Eysenck [1] noted that trait anxiety had no effect on digit span (a measure of WM, discussed later) in nine studies. Among those studies that did show significant effects, some demonstrated a positive relationship between trait anxiety and WM, while others showed a negative relationship or no relation at all. In order to explain these differences, the Processing Efficiency Theory (PET) posits that trait anxiety does not necessarily affect performance accuracy (effectiveness) in a task, but rather the speed and cognitive load (efficiency). In other words, for individuals who are predisposed to experiencing anxiety, the cognitive cost and speed of processing a task may be greater [5]. The theory proposes that anxious individuals allocate additional processing resources to implement compensatory strategies designed to improve their performance. According to Owens et al. [6], this advantage is possible for individuals with cognitive resources—such as high working memory, in their study—to compensate for or cope with the negative effects of anxiety. This is why the Attentional Control Theory (ACT), an extension of the PET, predicts that the repercussions would be more likely to manifest when cognitive functions requiring attentional control are engaged [7,8]. The ACT, aligning with Miyake et al.’s model [9], specifically focuses on three major executive functions (EFs): inhibition, updating, and shifting. These functions are crucial for guiding, controlling, and regulating actions and behaviors which are essential to learning and daily tasks [10]. Initially, the Attentional Control Theory (ACT) posited that anxiety would specifically impact inhibition and flexibility, but not updating [11]. This function, defined as “the constant monitoring and rapid addition/removal of contents from working memory” [12] (p. 9), was thought to be sensitive to anxiety only in threatening situations (e.g., using anxiety-inducing words as stimuli). However, Gustavson and Miyake [13] proposed that working memory updating is often assessed in terms of memory span (effectiveness) while the effects of anxiety might be more pronounced in response times (efficiency). Utilizing a computerized task for updating memory, their work confirmed anxiety’s impact on efficiency rather than effectiveness, in line with the ACT. The neutral nature of the words used in their study also does not support the ACT’s proposal of an impact of anxiety on updating in threatening situations. Furthermore, the ACT suggests that it is necessary for the tasks used to involve attentional control in order for anxiety to have a significant and detrimental effect [11]. In this regard, Moran [14] highlights a more pronounced effect of anxiety on measures of complex span tasks in which simultaneous processing of the items to be remembered is preceded or followed by concurrent activity [15]. Simpler tasks, such as digit span, which do not involve simultaneous processing, would only rely on storage and repetition of the elements to be remembered, making them less sensitive to anxiety. This type of task has been extensively studied, but it has been gradually overshadowed by tasks considered to be more complex and more appropriate for assessing working memory. This leads us to consider the digit span task, a classic yet nuanced tool in cognitive assessment that is still widely used in neuropsychological test batteries. This task comprises multiple subtests, and some researchers or clinicians may consider the forward digit recall component as assessing short-term memory, while backward recall may require more attention, making it a complex span task reflecting both the phonological loop and the central executive of WM [16,17]. Schmeichel [18] reported that backward span was sensitive to reduced executive capacity following previous executive control efforts. He concluded that inhibition, used to test executive control, relied on an underlying capacity shared with information updating. However, St Clair-Thompson and Allen [19] conducted a comparative study of these two tasks and concluded that backward recall behaved more like a simple span task, with relatively minimal additional processing required only during the recall stage. This is consistent with similar findings [20]: backward and forward span reflect the same cognitive abilities. As suggested by Schmeichel’s study [18], adding a sequencing task to the digit span (e.g., ascending order sequencing) could make the total score more representative of complex WM, but evidence does not support this [21]. Considering the observations made by Gustavson and Miyake [13] about the incomplete assessment of working memory tasks when limited to memory span, and its frequent use in clinical settings, it is justified to continue evaluating the digit span task from various angles to analyze the impact of anxiety on this task.

### 1.2. Attentional Control Theory and Measurement

At first glance, simple tasks may seem less relevant for research protocols focusing on the evaluation of the ACT. However, the ACT has primarily been studied at an algorithmic level rather than a reflexive level [2,22]. Toplak et al. [23] sought to understand and explain the existing differences in measurements of executive functions (EFs). Drawing on Stanovich’s framework [24,25], they propose that the questionnaire-based assessment of EFs may reflect an individual’s goals, beliefs associated with those objectives, and the selection of reasoned actions guided by those objectives and beliefs (referred to as the reflexive level). Tasks assessing EF performance allow for the observation of underlying information processing mechanisms, including processes like information coding, perceptual encoding, long-term memory utilization, etc. (referred to as the algorithmic level). Both levels of thinking are likely to be engaged by the compensatory strategies of the ACT when a specific goal is given.

A study by Cécillon et al. [26] has shown some initial interesting findings. They suggest an impact of the reflexive level on the amplification of trait anxiety, which would explain the consequences of problematic behaviors related to EFs and academic outcomes in adolescents. Although they did not measure EF performance, the authors concluded that overreliance on the reflexive level to compensate for performance in anxious individuals could lead to cognitive resource depletion, thus increasing the manifestation of these problematic behaviors as reported by parents in daily life. In this regard, Stanovich [25] emphasizes that both the reflexive and algorithmic levels are slow, require a great deal of attention, and interfere with our thoughts and actions. Compensatory strategies can manifest from both an algorithmic perspective—through a reorientation of information processing—and a reflexive perspective—through changes in goals or beliefs related to one’s performance. In other words, the ACT states that effects of anxiety on performance can be observed in certain tasks, such as demanding complex span tasks. Conversely, simple span tasks that require minimal attentional resources should not be affected by trait anxiety. However, the contribution of Stanovich’s theory suggests that the repercussions of anxiety could also be visible at a reflexive level, which may not be directly observable in the performance itself. Currently, ACT theorists propose that, if no performance differences are observed between anxious and non-anxious individuals, other indicators are available to account for the additional processing cost, such as an increased response time, heightened conflict monitoring after errors (detected by a high amplitude of the Error-Related Negativity signal), higher error rates, etc. [22,27]. Here again, the ACT considers behavioral indicators as if compensatory strategies only pertain to the algorithmic level.

### 1.3. Executive Function and Emotion Regulation

Previously, we discussed the involvement of anxiety in executive functions (EFs). In their literature review on EFs, Baggetta and Alexander [10] note that EFs are cognitive processes that also involve the socio-emotional domain. The most influential model that considers this domain is the one proposed by Zelazo and Cunningham [28]. They postulate the existence of distinct pathways that work together depending on the presence or absence of emotions in information processing. “Cool” executive functions are used when individuals face abstract and decontextualized problems. “Hot” executive functions are engaged in tasks requiring emotion regulation to achieve a goal or when the individual is actively involved and motivated in the task. The ACT predicts that, if the task is non-demanding or lacks clear objectives, anxious individuals will have little motivation to use attentional control mechanisms. However, for demanding tasks with specific objectives, the level of motivation would be high [2,6]. It is in these cases that anxious individuals would extensively employ compensatory strategies. According to this hypothesis, the tasks that are most sensitive to anxiety are, by extension, those that engage “hot” executive functions and emotion regulation. Compensatory strategies may be represented, in part, at a reflexive level, by conscious attempts to reduce unpleasant emotions experienced during the task to improve performance. This field was extensively investigated on the basis of conscious emotion regulation strategies (ERS) by developing the Cognitive Emotion Regulation Questionnaire (CERQ) [29]. In this questionnaire, certain strategies are considered adaptive, such as Refocuson planning or Acceptance, and others are considered maladaptive, such as Self-blame or Catastrophizing. While this binary distinction has been criticized and updated by Ford et al., it remains useful for highlighting thought patterns that may influence the maintenance of anxiety or performance in tasks involving executive functions [30]. ERSs have consistently been associated with psychopathology, specifically anxiety. The meta-analysis by Aldao et al. revealed that maladaptive ERSs (Rumination, Avoidance, and Suppression) were associated with greater psychopathology, while adaptive ERSs (Acceptance, Reappraisal, and Problem-solving) were associated with lower psychopathology across various psychological disorders [31]. Maladaptive ERSs showed a stronger association with psychopathology compared to adaptive ERSs, with the exception of problem-solving. Different associations were observed between mood disorders such as anxiety and depression, and externalizing disorders (substance use and eating disorders), suggesting that the use of ERSs may have different effects on behaviors or emotions. Significant associations between adaptive and maladaptive ERSs and symptoms of anxiety and depression were also described in another meta-analysis conducted with adolescents [32]. A Japanese meta-analysis examining the CERQ and its relation to anxiety (8 studies) and depression (16 studies) confirmed previous findings [33]. Some strategies yielded unexpected results, such as Blaming Others and Acceptance, which were positively associated with anxiety and depression. Although the direction of the relationship was as expected, Blaming Others had the smallest absolute value. Regarding Acceptance, Wilson suggests that it can be applied actively as a form of self-assertion or passively as a form of resignation [34]. Therefore, the sensitivity of the questionnaire, especially concerning this strategy, hinges on the individual’s interpretation of acceptance. Despite the instrument’s shortcomings and the simplistic binary framework for understanding emotion regulation strategies (ERSs), these strategies are probably significant in the interplay between anxiety and executive functions (EFs). McLaughlin et al. demonstrated that emotional dysregulation could be the cause of anxiety rather than the reverse [35]. ERSs may act upstream of anxiety. However, these conclusions should not be overgeneralized. While their study has robust internal validity, the questionnaires used limit the generalizability of their findings [26], especially in relation to our study. We suggest continuing to conduct correlations rather than regressions until further studies have been conducted which incorporate additional tools. Regarding the link between ERSs and Executive Functions (EFs), several studies have shown significant correlations between ERSs and EFs as assessed by parents [26,36]. These studies found that adolescents reporting the use of maladaptive ERSs (except for Blaming Others) were more likely to exhibit problematic behaviors related to EFs, as evaluated by the Behavior Rating of Executive Function (BRIEF) [37,38]. Conversely, adaptive ERSs were correlated with less problematic behaviors, except for positive refocusing and reappraisal. Most studies have focused on the influence of cognitive abilities on emotion regulation [39]. Studies on WM suggest that individuals with a low updating capacity may have depleted most of their executive resources, making it difficult for them to regulate their emotional experiences effectively [40,41]. In this regard, Barkus’s recent literature review [42] reveals that the increased rejection of maladaptive ERSs could be explained by a greater WM capacity. However, the results were more mixed regarding the influence of WM on the choice of adaptive ERSs [42]. In fact, the development of adaptive ERSs is not necessarily linked to greater EF capabilities [43,44]. For example, Veloso and Ty showed that training in emotional WM was associated with a decrease in trait anxiety, but it did not improve ERSs [45]. The authors argue that training individuals to divert their attention from emotionally salient stimuli and focus on task-relevant information may indeed impact some ERS processes, but not the ones examined in their study (Reappraisal and Suppression). These findings complement those of Pe et al. [40], who suggested that effective updating abilities can preserve cognitive resources for emotion regulation. Some researchers have also attempted to characterize the direction of the relationship between EFs and ERSs. One of the few studies that investigated the influence of emotion regulation on EFs provided evidence that inhibition, but not switching, was more strongly engaged during emotion regulation, leading to interference with the task [46]. Considering the ACT, this suggests that emotion regulation should be considered when studying the relationship between anxiety and EF performance. In this context, the use of working memory tasks such as the digit span might provide limited information about the link between anxiety, emotion regulation, and performance. N-back tasks can provide a wealth of information about how a person makes decisions and mobilizes their capacities. Meule [47] argues that the accuracy score and RT are not interchangeable and provide non-redundant information. Additionally, Meule suggests using omissions (not pressing a button) and commissions (making an error by clicking the wrong button), which would provide additional information on the participants’ decision-making. Therefore, the use of this type of working memory task employing emotional stimuli could be an opportunity to better understand the ACT in connection with emotion regulation strategies. The use of maladaptive ERSs could create an interference effect in the task, which could explain the lower efficiency among anxious individuals.

### 1.4. Metacognitive Beliefs

Wells [48] proposed a theory and therapy aimed at addressing thoughts that may exacerbate or maintain mood disorders, including anxiety. According to Wells, there is a metacognitive thinking mode that leads individuals to view mental events, perceptions, or emotions as separate from themselves. In contrast, the object mode encourages individuals to see these elements as integral to themselves [48]. These specific thinking styles influence individuals to use strategies to regulate their thoughts and feelings. In the object mode, individuals adhere to certain metacognitive beliefs that perpetuate and exacerbate biased threat evaluation. For example, a positive belief in worry, such as “I need to worry in order to work well,” encourages vigilance toward threatening stimuli, while beliefs about thought control (Control factor), such as “If I did not control a worrying thought, and then it happened, it would be my fault,” prevent individuals from changing their perspective. It is worth noting that the latter example can significantly contribute to the use of maladaptive ERSs, such as Self-blame in the CERQ. Other negative beliefs, such as “My worrying could make me go mad,” are likely to increase feelings of danger significantly and persistently. The MetaCognition Questionnaire (e.g., MCQ-65) [49] was developed to assess these beliefs, as well as a lack of confidence in one’s cognitive resources and awareness of one’s thoughts. In adults, this scale has been highly effective in explaining the propensity to experience anxiety (explaining 83% of the variance) [50]. Some findings show that the subscale of negative metacognitive beliefs (MCneg) is consistently linked to various symptoms, including anxiety, in both clinical and non-clinical populations [50,51,52]. Similarly, the global scale and MCneg predict the use of maladaptive ERSs or emotional dysregulation in several studies with healthy individuals [53,54,55]. In contrast, positive metacognitive beliefs and the Consciousness scale have less pronounced effects than the other subscales [56,57,58]. The studies by Mansuetto et al. [59] and Laghi et al. [60] highlight that the Consciousness scale had an inverse relationship with emotional dysregulation. In other words, focusing attention on one’s thoughts was associated with better emotional regulation. Cécillon et al. [26] demonstrated in French adolescents that the Consciousness scale was associated with the use of both maladaptive and adaptive ERSs. The authors concluded that being aware of one’s thoughts prompts individuals to choose ways to regulate their emotions that may not necessarily be maladaptive. Like Sica et al. [61], they propose revising this subscale to emphasize the negative aspects of excessive and rigid consciousness to better represent the object mode advocated by Wells [48].

Ultimately, this new reflective variable has strong links with emotion regulation and anxiety. However, not all sub-scales of the MCQ have the same relationship with trait anxiety and ERSs, which will not necessarily affect information processing in the same way. Spada et al. [62] highlighted how the MCneg and Control factors create a cognitive gridlock, which produces even more worry. Preliminary evidence has been provided on the involvement of metacognitive beliefs in a difficulty to use executive functions [63], including working memory [64]. Therefore, metacognitive beliefs could play a role in the interference of information processing in working memory tasks.

### 1.5. Present Study

Given these different reflective variables—metacognitive beliefs and ERS—we believed that all tasks were likely to reflect the impact of anxiety on information processing efficiency. However, measurement did not necessarily occur at the behavioral level as proposed in the ACT. Eysenck et al. [2] described compensatory strategies at an algorithmic level, which is not independent of the reflective level [65]. The reflective level partly involved conscious thoughts and strategies that individuals might have had during the task. Many studies pointed in this direction without necessarily making the connection with the ACT [66,67,68]. Our study aimed (1) to extend the findings of Cécillon et al. [32] to an older, non-clinical population and (2) to observe the influence of the reflective level on the algorithmic level in the ACT.

Regarding the first objective of this article, we hypothesized that similar links would be found between the reflective variables. The network analysis in the principal article showed a strong intertwinement between anxiety, emotion regulation strategies, and metacognitive beliefs, with strong correlations among them. Given the regularities observed between these variables in different age-variable samples [31,32,51], we expected to observe similar correlations in an older, non-clinical population. We performed analyses similar to those of Cécillon et al. [26].

Regarding the second objective, the ACT predicted that compensatory strategies would be mobilized during the task, which would explain why anxious individuals might show lower efficiency without necessarily diminishing their effectiveness. We expected that reflective variables would incur an additional information processing cost, manifesting in longer response times in the n-back and an increased number of strategies used during the digit span. Thus, maladaptive metacognitive beliefs and emotion regulation strategies were expected to predict the participants’ efficiency in working memory tasks as much as trait anxiety. Conversely, the performance (standard score in digit span and accuracy in n-back) of the subjects was not expected to be influenced by these variables. Since the support is emotional and the difficulty of the task has been noted as high by the authors of the n-back [40], we expected the effects of the reflective variables to be more pronounced on the n-back task compared to the digit span task. According to the authors, the correlates vary between these two pieces of information, and each of them provides complementary data. Consistent with the ACT, we expected that trait anxiety might explain longer response times in the n-back task with repercussions on performance. We also explored its impact on commissions and omissions and their links with other reflective variables.

Finally, this study provided an opportunity to exploratorily investigate the links between the n-back and digit span tasks, which presented different levels of complexity and very different content (emotional words vs. numbers). To our knowledge, these tasks had never been linked in detail by categorizing the types of errors made in the n-back or by measuring the strategies used during the digit span task. At a minimum, we expected to observe correlations between these two tasks since they both required retention and recall processes. Meule [47] suggested that omission and commission errors in the n-back task would provide additional information on the participants’ decision-making. Making an error can be due to multiple reasons such as interference. In this case, preceding words with different emotional valences can create proactive interference and lead to commission errors due to a lack of inhibition or impulsivity. Conversely, the subject may have difficulties in retrieving information, resulting in omission errors due to retroactive interference. An additional error could be an emotional salience bias. Words with positive or negative emotional valence may have different emotional salience, meaning that they can attract the subject’s attention more. Numerous studies on trait anxiety have shown the presence of attentional bias towards negative information. Errors can also be caused by WM overload, which can result in confusion (commission) or forgetting (omission). Given the limited information we have on digit span sequencing and this emotional n-back task, we will take advantage of the concurrent use of these two WM tasks to examine correlations between different variables, detailing the scores obtained for forward, backward, and ascending order series.

## 2. Materials and Methods

### 2.1. Sample

The study initially included 126 students, but after excluding 9 for incomplete tests, 4 due to being non-native French speakers, 1 due to a neurological operation, and 2 for misunderstanding task instructions, the final sample comprised 110 students aged 18–28 (M = 20.127, SD = 2.103), with 67.27% being female. Most were from Lyon 2 University (68.18%) and Catholic University of Lyon (25.45%). The sample included psychology students and was predominantly comprised of undergraduates (90%), while 10% were in master’s programs. The study included 11 reported cognitive or affective characteristics. The University Grenoble Alpes ethics committee approved the study, and all participants gave informed consent (CERGA-2022-25).

### 2.2. Procedure

The study was conducted over six months, spanning from November 2022 to April 2023. Recruitment of participants took place on campus, with all individuals volunteering without receiving any incentives or course credits. The participants engaged in the digit span task, based on the Wechsler Adult Intelligence Scale—4th Edition (WAIS-IV) [69], facilitated by a researcher, as well as a computerized emotional n-back task, which was modified from the work by Pe, Raes, and Kuppens [40]. Sessions averaged 25 min in length. Following the session, participants were asked to fill out questionnaires online at their convenience, ensuring all were completed within a month of the experimental tasks. To minimize order effects, the order in which participants performed the tasks was alternated: the digit span task was initially administered 55 times, while the n-back task was administered 57 times.

### 2.3. Measures

#### 2.3.1. Emotional n-Back

Participants undertook the emotional n-back task, designed to assess the updating of emotional information. This particular task was modified from the version developed by Pe, Raes, and Kuppens [40], who adapted their version from the original by Levens and Gotlib [70]. Unlike the original, which might have used faces or other stimuli, Pe and colleagues [40] opted for words as the stimuli in their adaptation. The materials utilized in their study were translated from Dutch to French, employing a back-translation method to ensure accuracy and fidelity in the translation process. When there were doubts about the translation, we relied on the Affective Norms of English Words list [71] originally used. Like Pe et al. [40], we listed the 47 positive words and the 49 negative words in a table, describing their valence, number of letters, and number of syllables, see Supplementary Materials [72]. One term was updated: “Malaria” was changed to “COVID”. The task consisted of 24 training trials and 96 actual trials, divided into 4 blocks of 24 trials. The first two trials of each block were not scored, leaving 88 trials for our analysis. Specifically, each trial involved the presentation of an emotional word in the center of the screen for 500 ms, followed by an inter-trial interval of 2500 ms. Participants were instructed to press the “1” or “2” key if, respectively, the valence of the current word matched the valence of the word two trials ago (match) or if the valence did not match (non-match). There were 44 match trials, equally divided between negative and positive valence stimuli (22 trials each), and 44 non-match trials (21 trials were positive valence stimuli, meaning that the current stimulus was positive but the stimulus two trials later was negative). Pe et al. [40] used average accuracy scores for all trials as the main measure rather than response time due to the difficulty of the task. To improve precision, we also calculated commission errors (pressing the incorrect key) and omission errors (not pressing any key).

#### 2.3.2. Digit Span Task

Participants completed three subtests of the digit span task: recalling number series in the same order (forward), in reverse order (backward), and in ascending order (sequencing). Numbers were presented at one per second, and participants repeated them, following procedures in the administration manual [69]. Raw scores, maximum digits recalled, and age-relative standard scores were recorded. At the end of the task administration, participants’ strategies (e.g., subvocal repetition, visualization) were noted and scored, with a total strategy score calculated for each. Intuitive recall without a specific strategy was scored as 0.

#### 2.3.3. Online Questionnaires

The questionnaire included consent forms and questions about age, sex, and education level. The last 4 digits of their mobile phone number were also requested to link the responses to the results from the laboratory tasks. We then administered three self-reported questionnaires.

##### Trait Anxiety

Trait anxiety was evaluated using the French adaptation of the State Trait Anxiety Inventory version Y (STAI) [73] by Gauthier and Bouchard [74]. This tool is designed to measure both state and trait anxiety, but in this case, only the trait anxiety component was utilized. The assessment comprises 20 items, each rated on a 4-point Likert scale, with options ranging from 1 (almost never) to 4 (almost always). The scale also incorporates reversed items to ensure comprehensive assessment. In the French version, the reliability of the scale, as indicated by Cronbach’s alpha, averaged around 0.90.

##### Emotion Regulation Strategies

Emotion regulation strategies were evaluated with the Cognitive Emotion Regulation Questionnaire (CERQ) [29], utilized in its validated French edition [75]. The CERQ is a self-administered questionnaire designed to identify nine cognitive strategies individuals employ to manage emotions following negative or unpleasant events. It consists of 36 items, each rated on a Likert scale from “almost never” (1) to “almost always” (5). These items are distributed across the nine distinct strategies for regulating emotions: Acceptance (A; “I think I should accept that it happened”), Positive Refocusing (PR; “I think about more pleasant things than what I have experienced”), Planning (P; “I think of a plan regarding the best way to handle it”), Positive Reappraisal (PR; “I think I can become a stronger person because of what happened”), Putting into Perspective (PP), Self-blame (SB; “I feel that I am to blame for what happened”), Rumination (R; “I keep thinking about how awful the situation was”), Catastrophizing (C; “I often think that what I experienced is the worst thing that can happen to someone”), and Blaming Others (BA; “I feel that ultimately others are to blame for what happened”). The questionnaire was validated for use in a demographic ranging from 13 to 19 years old. Participants are guided to contemplate their thought processes during encounters with negative or unpleasant events. In its French adaptation, the reliability of the questionnaire, as measured by Cronbach’s alphas, spans from 0.68 to 0.87 across all factors.

##### Metacognitive Beliefs

Metacognitive beliefs were assessed using the Short MetaCognition Questionnaire (MCQ-30) [76], which was used in its validated French version [77]. The questionnaire is composed of 30 items, equally divided among five distinct factors: Positive metacognitive beliefs (MCpos factor), Beliefs about uncontrollability and danger (MCneg factor), Beliefs about cognitive competence (Lack of Confidence factor), Negative beliefs about thoughts related to superstitions, punishment, and responsibility (Control factor), and Cognitive self-consciousness (Consciousness factor). In the questionnaire, participants rate statements using a 4-point Likert scale that goes from “Not at all agree” to “Completely agree.” Scores for each factor within the questionnaire vary from 6 to 24, leading to an overall minimum score of 30 and a maximum of 120. For the French adaptation, the internal consistency of the questionnaire, as indicated by Cronbach’s alpha coefficients, ranges from 0.72 to 0.93 across all factors.

### 2.4. Data Analyses

We conducted numerous additional analyses to replicate the findings of Cécillon et al. [26]. To maintain the primary focus of this article, we opted to include these analyses along with the discussion of their results in the Supplementary Materials. This is made available at Mendeley Data website, reference number [72], with the complete dataset. These results demonstrate differences in the expression of anxiety, the use of emotion regulation strategies, metacognitive beliefs, and working memory utilization between boys and girls. Hence, we controlled for sex in all our analyses. In anticipation of the numerous analyses planned in our study, we estimated the sample size using the Free Statistics Calculators website [78]. We entered the value of 0.15 for the anticipated effect size (*f*^2^), 0.8 for the desired statistical power level, and 5 as the number of predictors. The alpha risk was set at 0.05. This resulted in a required sample size of 91. Before testing our hypotheses, we analyzed the continuity of variables. All the variables exhibited skewness [−0.50; 0.22] and kurtosis [−0.96; −0.04] indices within an acceptable range to conduct parametric tests, as these indices fell between −2 and +2 [79]. It is important to note that the total standard score of the digit span task was close to the average, but some values could be considered as deficient or exceptionally high. We chose to keep all the data, considering it representative of the student population in our sample.

## 3. Results

### 3.1. Trait Anxiety

#### 3.1.1. ERS and Trait Anxiety

A correlation matrix was conducted between trait anxiety and emotion regulation strategies. Sex was controlled for in these correlations. Maladaptive ERSs showed a strong, positive, and significant correlation with trait anxiety, as did the sub-scales Self-blame, Rumination, and Catastrophizing (respectively, *r* = 0.594, *r* = 0.459, *r* = 0.290; *p* < 0.001), except for Blaming Others (*r* = −0.072). On the other hand, adaptive ERSs showed weaker and non-significant correlations with anxiety. Only the sub-scales Positive Refocusing (*r* = −0.231, *p* = 0.016) and Positive Reappraisal (*r* = 0.241, *p* = 0.012) were significantly, but weakly, associated with trait anxiety compared to Self-blame and Rumination.

#### 3.1.2. Metacognitive Beliefs and Trait Anxiety

The linear regression analysis was conducted to assess the effect of metacognitive beliefs on the overall anxiety levels of participants, with adjustments made to account for the variable of participants’ sexes. The bootstrapping was based on 5000 replicates. It revealed that a high metacognition score significantly predicted the variance in the total anxiety (β = 0.462, SE = 0.061, *p* < 0.001, t = 7.589, 95% CI [0.34, 0.58]). Another linear regression showed that the MCneg factor largely accounted for this prediction (β = 1.431, SE = 0.173, *p* < 0.001, t = 8.251, 95% CI [1.09, 1.77]), followed by the Control factor (β = 0.486, SE = 0.169, *p* = 0.005, t = 2.868, 95% CI [0.15, 0.82]). The MCpos (β = −0.235, SE = 0.153, t = −1.539, 95% CI [−0.53, 0.07]), Lack of Confidence (β = 0.209, SE = 0.140, t = 1.487, 95% CI [−0.07, 0.49]), and Consciousness factors (β = 0.198, SE = 0.167, t = 1.188, 95% CI [−0.13, 0.53]) weakly and non-significantly predicted trait anxiety.

### 3.2. Correlations between Metacognitive Beliefs and Emotion Regulation Strategies

A Pearson correlation matrix was conducted to determine the strength of the associations between metacognitions and ERSs. Maladaptive ERSs were positively correlated with all subscales of the MCQ. However, the significance threshold was not reached for positive metacognitive beliefs. The strongest correlations were found between negative beliefs (*r* = 0.458, *p* < 0.001), the Control factor (*r* = 0.410, *p* < 0.001), and the Consciousness factor (*r* = 0.342, *p* < 0.001). There was a weaker correlation between Lack of Confidence and maladaptive ERSs (*r* = 0.218, *p* < 0.05). Adaptive ERSs showed weak and non-significant negative correlations with negative metacognitions, the Lack of Confidence factor, and the Control factor. Positive metacognitions were positively correlated (*r* = 0.121), but the correlation was not significant. However, the consciousness factor showed a significant positive correlation with adaptive ERSs (*r* = 0.215, *p* < 0.05).

### 3.3. Relation between Working Memory Tasks

Before proceeding with further analyses, we present the descriptive statistics of our variables (Table 1). The accuracy score of the n-back task falls within the range of Pe et al.’s study (Study 1: M = 0.59 and SD = 0.13; Study 2: M = 0.67 and SD = 0.14) [40]. The commission scores were calculated by summing all errors per participant and dividing the total by the number of trials. The omission scores were calculated by summing the trials with no response and dividing by the total number of trials. A Student’s paired-samples t-test revealed that the commission scores were significantly lower than the omission scores, indicating that the participants made more omission errors than commission errors (t = 7.269; df = 109; *p* < 0.001).

We also separated the commissions and omissions made due to proactive interference on sequences alternating emotional valences (negative word—positive word—negative word or positive word—negative word—positive word). We thus isolated the 24 responses containing this type of sequence from the other 88. For each participant, we calculated an average for the number of commission errors and omission errors, which we named Emotional Total Commission error (ETC) and Emotional Total Omission error (ETO). We also subdivided the 24 sequences by differentiating between positive sequences (positive word—negative word—positive word) and negative sequences (negative word—positive word—negative word) to assess potential emotional salience biases. For each participant, we calculated an average for the number of negative commission errors (NCEs), positive commission errors (PCEs), negative omission errors (NOEs), and positive omission errors (POEs).

We conducted paired-samples *t*-tests to determine if there were differences between ETCs and ETOs compared to total commission errors and total omission errors. Participants had significantly more ETCs than commission errors (t = 4.936; df = 109; *p* < 0.001). In other words, sequences that contained alternating emotional valence words resulted in more commission errors than other sequences. Conversely, participants had more omission errors than ETOs (t = −7.647; df = 109; *p* < 0.001). They were therefore more likely to not respond during sequences where emotions were not alternated.

We conducted a correlation matrix to analyze the relationships between the two tasks. For the n-back task, the commission and omission errors were strongly and significantly correlated with task accuracy (Table 2). Each of these errors showed an inverse relationship with the response time (RT). Omissions significantly increased the RT, while commissions tended to decrease the RT (*p* = 0.081). For the digit span tasks, correlations were very strong and significant with the total score. However, the correlations between the two tasks were weak but significant. The number of strategies employed during the tasks (Strategies) was not correlated with the total score or the digit span tasks. In terms of correlations between the tasks, the commission errors were correlated with all indicators of the digit span tasks. The weakest correlations were observed with the forward task and Strategies. Although, to a lesser extent, the task accuracy was also correlated with all tasks and the total score, except for Strategies. Lastly, the RT was correlated with the total score and the backward task. The correlation with the Sequencing task did not reach the threshold of significance (*p* = 0.90).

### 3.4. Relation between Working Memory Tasks and Main Variables

A Pearson correlation matrix (Table 3) was conducted to assess the relationships between our main variables while controlling for the effect of sex. Metacognitions were correlated with maladaptive ERSs and STAI values. Higher levels of metacognition in students were associated with a higher trait anxiety and greater use of maladaptive ERSs. Metacognitive beliefs were also correlated with the RT and omission scores in the n-back task. Participants with stronger metacognitive beliefs had longer RTs and higher rates of omission. Maladaptive ERSs were correlated with trait anxiety, RT, and the number of strategies employed during the digit span task. In other words, individuals using maladaptive ERSs were more likely to have a higher trait anxiety and longer RTs, and employ more strategies during the digit span task. More precisely, Rumination and Catastrophizing were positively and significantly correlated with the number of strategies employed (respectively, r = 0.255; *p* = 0.008 and r = 0.227; *p* = 0.018). Self-blame and Blaming Others showed no correlation. A significant negative correlation was found between maladaptive ERSs and commission scores. Trait anxiety was positively correlated with the RT in the n-back task but was not correlated with other indicators of working memory performance. However, trait anxiety had close-to-significant correlations with adaptive ERSs (*p* = 0.079), omission scores (*p* = 0.052), and emotional omission scores (*p* = 0.064). Omission scores also had close-to-significant correlations with maladaptive ERSs (*p* = 0.060) and adaptive ERSs (*p* = 0.052).

Metacognition showed a strong positive and significant correlation with omission scores. ERSms were correlated with the total omission score, specifically with NOEs. STAI was also significantly and exclusively correlated with NOEs. The correlations of the ERSas were all non-significant but showed an inverse relationship with the ERSms for omission scores. Finally, only the ERSms exhibited a significant negative correlation with NECs. In other words, individuals using ERSms made fewer commission errors on sequences involving alternating emotional valences that required a negative response. As before, to understand the inappropriate strategies that were involved in the correlation with commissions, we conducted a new correlation for each of the strategies. The results show that Catastrophizing and Rumination were negatively associated with negative commissions (respectively, r = −0.182; *p* = 0.059 and r = −0.226; *p* = 0.018). Self-blame and Blaming Others showed no correlation.

Several regression analyses were conducted to determine the influence of reflective variables (Anxiety, ERSa, ERSm, MCQ) on the n-back task, specifically on the RT and accuracy, and on the digit span task, focusing on the standard score achieved and the number of strategies used during the test. Bootstrapping was conducted based on 5000 replicates. The regression analyses showed no significant effects of the reflective variables on the overall accuracy in the n-back task or on the standard score in the digit span task. However, only ERSms significantly and positively predicted the RT in the n-back task (β = 9.184, SE = 3.972, *p* = 0.023, t = 2.312, 95% CI [1.31, 17.06]) and the number of strategies used (β = 0.042, SE = 0.012, *p* < 0.001, t = 3.547, 95% CI [0.018, 0.065]). The use of ERSms increased the RT. These ERSms predicted an increase in the number of strategies employed in the digit span task. Given the significant conceptual differences between the subscales of metacognitive beliefs and their unequal implications for anxiety, we conducted regression analyses considering each of the subscales and their effects on the same dependent variables. Only the Lack of Confidence factor significantly predicted a longer RT (β = 14.99, SE = 6.204, *p* = 0.017, t = 2.417, 95% CI [2.69, 27.30]). The significance threshold was retained after bootstrapping based on 5000 replicates (*p* = 0.024). No other effects of the MCQ subscales were found.

**Figure 1:**
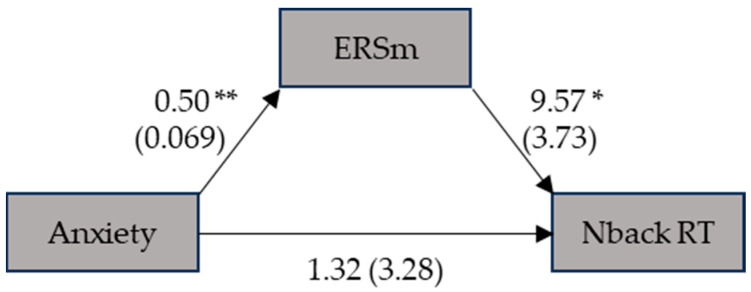
Mediating role of emotion regulation strategies in the relationship between anxiety and response time in cognitive tasks. ERSms = maladaptive Emotion Regulation Strategies; n-back RT = n-back Response Time. Delta method standard errors, bias-corrected percentile bootstrap confidence intervals, 1000 samples. Background confounder: sex. * *p* = 0.010; ** *p* < 0.001.

Finally, we conducted two mediation analyses to assess the influence of anxiety on the RT in the n-back task and strategy use in the digit span task, using ERSms as a mediator. The first mediation revealed no direct influence of anxiety on the RT in the n-back task (β = 1.316; SE = 3.284; *p* = 0.69; CI [−5.88, 7.64]) (Figure 1). In contrast, the indirect effect of anxiety on the RT through ERSms was significant (β = 4.787; SE = 1.981; *p* = 0.016; CI [1.38, 9.34]). Ultimately, the total effect became significant (β = 6.103; SE = 2.78; *p* = 0.028; CI [0.25, 12.03]), highlighting the effect of anxiety, which increased the RT through the mediation of ERSms.

**Figure 2:**
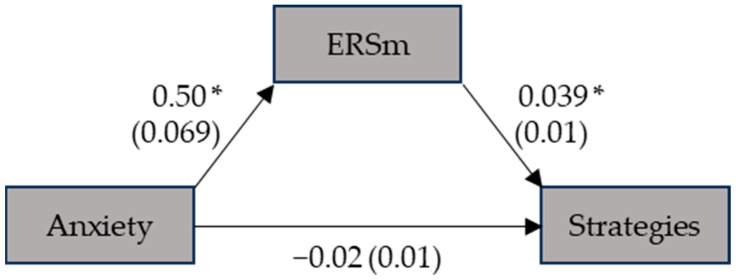
Mediating role of emotion regulation strategies in the relationship between anxiety and strategies used in digit span task. ERSms = maladaptive Emotion Regulation Strategiese; n-back RT = n-back Response Time. Delta method standard errors, bias-corrected percentile bootstrap confidence intervals, 1000 samples. Background confounders: sex. * *p* < 0.001.

Regarding the second analysis of strategies used during the digit span, the direct effect of anxiety was not significant, suggesting that anxiety does not directly influence strategy use in a straightforward manner (β = −0.018; SE = 0.010; *p* = 0.062; CI [−0.04, 0.00]). This result indicates that the level of trait anxiety does not have a direct, linear impact on the number of strategies employed during the task. However, the indirect effect of anxiety on strategy use, mediated by ERSms, was found to be significant (β = 0.019; SE = 0.006; *p* = 0.002; CI [0.01, 0.03]) (Figure 2). When considering the total effect of anxiety on strategy use, encompassing both direct and indirect pathways, the effect was not significant (β = 0.00; SE = 0.009; *p* = 0.908; CI [−0.02, 0.02]).

## 4. Discussion

The first objective of the present study was to verify and generalize the findings from Cécillon et al.’s [26] study regarding the reflective dispositions—metacognitive beliefs and ERS—that may influence trait anxiety levels. The second objective was to observe the impact of these dispositions on WM performance, reflecting the algorithmic level. The ACT proposes that anxious individuals should use compensatory strategies during tasks to adjust their performance, which would impact their processing efficiency. This additional cost has been consistently described at the algorithmic level in terms of behavioral measures [2], but has not been extensively studied to understand the influence of the reflective level on this aspect, in line with Stanovich’s theory [24]. This study aimed to shed new light on the ACT in relation to this aspect. Finally, the last objective of this study was to explore the connections between the digit memory task and the emotional n-back task, while delving deeper into the specific measures of each. To facilitate reading, we have organized this discussion by separating the levels of information processing: reflective (objective 1) and algorithmic (objective 3). Then, we concluded with the interaction between the two levels (objective 2).

### 4.1. Trait Anxiety, Metacognitive Beliefs and ERS (Reflexive Level)

Our data replicate and confirm similar results to those of Cécillon et al. [26]. Metacognitive beliefs, maladaptive ERSs, and trait anxiety were strongly interrelated to an equivalent extent. Specifically, trait anxiety was found to be correlated with the subscales of maladaptive ERSs, except for Blaming Others. Additionally, adaptive ERSs were weakly correlated with trait anxiety, with only two of the subscales, positive refocusing and reappraisal, showing significant correlations, while the others exhibited no correlation. The absence or weakness of correlations between adaptive ERSs and Blaming Others has been noted in several studies [31,32,33,80,81]. We do not reiterate all the conclusions and proposed solutions from Cécillon et al. [26] here, as the aim of this study was to extend their findings to a different population. The main difference in our results was the presence of correlations between anxiety and the positive refocusing and reappraisal subscales. Aldao and Nolen-Hoeksema [82] propose that adaptive ERSs are more context-dependent than maladaptive ERSs. They observe greater inter-situational variability in the implementation of acceptance and problem-solving. This increased variability suggests that the use of adaptive strategies is influenced by a more flexible evaluation of different contextual situations. Instead of a random approach where individuals non-systematically and uniformly try different adaptive strategies, they appear to adopt a more targeted and tailored approach based on specific variations in each context. Therefore, it is entirely normal in our study to not observe correlations between all adaptive ERSs, such as acceptance, and anxiety. Conversely, the implementation of maladaptive ERSs showed a low variability, suggesting their comparable usage across situations. This explains the more consistent results observed in most studies.

Our findings are consistent with those of Cécillon et al. [26] and the previous literature on metacognitive beliefs, maladaptive ERSs, and trait anxiety. Metacognitive beliefs were found to predict participants’ trait anxiety. The MCneg and control subscales were the only ones that significantly predicted anxiety, with a larger effect size for MCneg. The other three factors did not show significant associations. Nordhal et al. [50] also reported a similar correlation between MCneg, control, and anxiety, but they found additional weaker yet significant correlations for the other factors. Given the replicability of our results in adolescents and young adults, these differences could be partly explained by cultural bias and differences in the interpretation of certain beliefs. Interestingly, the MCpos factor negatively predicted anxiety. Like the consciousness factor, MCpos was correlated with both adaptive and maladaptive ERSs. However, the correlation with maladaptive ERSs, like the consciousness factor, was not significant but proportionate. These results align with Cécillon et al. [26], who found a significant correlation between MCpos and maladaptive ERSs. This suggests that considering worry as something that could help predict the future (MCpos) is not necessarily negative or likely to amplify anxiety. Like the consciousness factor, MCpos may be a way to direct behaviors toward adaptive or maladaptive ERSs. However, a recent meta-analysis on healthy adults and individuals with psychopathology revealed that the combined effects were reliable for all four scales of the MCQ, but the positive metacognitive beliefs subscale was unstable or even nonsignificant [83]. Benedetto et al. [57] suggested that positive metacognitive beliefs could be considered an effective coping strategy. In our previous study with adolescents, we concluded that they did not seem to be effective since they were positively correlated with maladaptive ERSs. The current findings partly support this hypothesis by indicating that positive metacognitive beliefs can play a determining role, albeit less important than the consciousness factor, in individuals’ inclination toward the use of adaptive or maladaptive ERSs. The changes observed in the MCQ may be due to cultural factors as well as age-related factors. Irak [58] demonstrated a significant age effect on MCpos. Significant differences observed between different school years suggested that the development of MCpos could be influenced by factors related to the cognitive and emotional development of children and adolescents. Similarly, a study on French-speaking adolescents [84] showed that the MCneg, MCpos, and consciousness factors significantly increased between the ages of 13 and 17. However, it is important to note that the age effect in Irak’s study, although significant, was relatively small, suggesting that other factors may also contribute to the variation in MCpos beyond age alone. Unfortunately, studies specifically examining age and other factors related to the MCQ that may explain such variations are limited. These findings should be considered when interpreting future studies using the MCQ on healthy samples. It is likely that, in samples with emotional disorders, the Consciousness scale would be correlated with psychopathology, as predominantly maladaptive emotion regulation strategies would be used. As suggested by Sica et al. [61] and Cécillon et al. [26], it may be beneficial to rephrase the items of this scale to highlight a more negative aspect of excessive and rigid consciousness, which would make the questionnaire more discriminant.

### 4.2. Working Memory (Algorithmic Level)

#### 4.2.1. Emotional n-Back

Regarding the n-back task, our results were reassuring, as the average accuracy rate was equivalent to those reported in two studies by Pe et al. [40] that also utilized the task. The low accuracy rate indicates that the task was highly challenging compared to similar tasks [41,70] which achieved accuracy scores above 80%. Meule [47] emphasized the importance of considering multiple aspects of the n-back task, including the accuracy, omission, and commission rates, as well as the response times, as they can provide different insights. Our findings support this perspective, revealing significant associations between the omission/commission scores and accuracy, although no significant relationship was found between the omission and commission scores themselves. Additionally, the relationships between the omission and commission scores and the response times (RT) were different. The commission errors showed a negative correlation, although not significant (*p* = 0.081), suggesting that these errors are not necessarily associated with inhibition deficits or impulsivity. In contrast, the omissions were positively and significantly correlated with the RT, indicating that participants took longer to respond when they omitted a response. These results highlight that the commission score is minimally associated with the response time compared to omission errors in our study. Commission and omission errors provide different and non-redundant information. We also hypothesized that proactive interference might lead to impulsive responses in participants. However, our results showed that the response time (RT) was minimally correlated with commission errors, challenging this hypothesis. This suggests that commission errors are not necessarily related to inhibition deficits or impulsivity but may be influenced by other factors. Furthermore, when specifically examining sequences where positive and negative emotional valences alternate, we found that the alternation of emotional valences was associated with more frequent and regular commission errors. This observation suggests the presence of emotional interference in the decision-making process, where emotional stimuli can disrupt performance and increase commission errors. It is possible that this emotional interference captures participants’ attention more strongly and leads them to make more errors when processing contrasting emotional stimuli. This interpretation may explain the higher frequency of total omission errors compared to emotional omission errors. The emotional alternation of responses may increase the cognitive load and engage participants’ attentional resources more, thereby facilitating the production of responses. In contrast, in sequences without emotional alternation, where emotional valences remain constant, the cognitive load and attention may be lower, increasing the risk of total omission errors. These findings highlight the complexity of the interactions among emotions, attention, and decision-making. Commission errors may be influenced by emotional interference, while omission errors may be influenced by the emotional salience of stimuli. Further research is needed to ensure the validity of these conclusions.

#### 4.2.2. Digits Span

Our results revealed strong positive correlations between the three tasks of the digit span from the WAIS and the total score. This indicates that a high performance in each individual task of the digit span is generally associated with a higher total score. However, it is important to note that the three tasks of the digit span showed weak correlations with each other. This suggests that the specific skills required to succeed in each task may differ, despite all of them sharing a component of digit memory. The specific correlations reveal that the backward task is strongly correlated with the forward task, confirming the findings of Jaeggi et al. [85]. This suggests a close relationship between these two tasks, likely limited to the recall phase [19]. However, the correlations remain weak, indicating the presence of distinct processes beyond this phase. Additionally, the backward task also shows relatively high correlations with the sequencing task. However, the sequencing task and the forward task have the weakest correlation among the three tasks, implying that they may be more influenced by specific and distinct factors. Some researchers consider the digit manipulation tasks (backward task) as an active component of WM, while the forward task is seen as more passive [16,17]. Other researchers consider the two tasks as similar with minimal differences [19,20]. At this stage, our data on the digit span alone do not allow us to come to a conclusion about the involvement of these three tasks in WM. Nevertheless, they suggest differences in the processes involved in each task. For example, Wisdom et al. [86] showed that the digit repetition sequence was inherently unique, as its age-related dispersion pattern behaved differently from the forward and backward tasks. It seems to provide distinct information that has not yet been fully understood. Additionally, Lumpkin and Sheerin [87] demonstrated that the digit span sequencing task was as relevant as the backward task in predicting neurocognitive impairments [88,89]. The specific factors responsible for these differences are not yet fully understood, and further research is needed to explore and identify them. One possible explanation that we propose is that the digit span sequencing task may more strongly engage and rely on the “mental number line” [90]. This mental representation of numbers is organized linearly in our minds. When performing the sequencing task, we not only have to remember the presented sequence of numbers but also order them in a certain sequence based on their respective positions. This requires a linear mental representation of numbers, that is, the ability to mentally place the digits in a sequential order. In contrast, the other two tasks may not rely as much on this cognitive representation as they do not require the same level of linear organization of numbers in WM. Thus, the use of the mental number line in the digit span sequencing task could be a key factor contributing to the observed variations between the tasks. This is a speculative hypothesis that warrants further investigation to substantiate and validate this idea. To partially address the question of the specificity of the mechanisms involved in each task, a comparison with the emotional n-back task could provide further insights. Furthermore, our study focused on the reflective level (conscious processes leading to decision-making based on beliefs and goals) and its impact on WM tasks. This perspective adds additional elements to understand the underlying processes involved in performing these tasks as will be discussed below.

#### 4.2.3. Digit Span and Emotional n-Back

Regarding the links with the digit span task, the accuracy score was correlated with all variables of the digit span, except for the number of strategies employed during the task. The strongest correlation was with the total score of the digit span, followed by the Sequencing task, Backward task, and then the Forward task. A previous study found correlations between the digit span and accuracy on various n-back tasks using auditory, visuospatial, and mixed (simultaneous auditory and visuospatial) materials [85]. The correlations they reported were like ours (0.21 to 0.30), which aligns with the results of the meta-analysis by Redick and Lindsey [91] on this topic. Interestingly, these correlations were only significant for 3-back tasks, indicating a high cognitive load. This confirms that the n-back task used in our study had a high cognitive load. Redick and Lindsey [91] propose that both the backward digit span task and the n-back task require temporal reorganization of information. In the backward task, participants need to update the position of elements in memory, while in the n-back task, they need to modify the position of previously encoded elements. According to them, this similarity in the reorganization process may explain the stronger correlation between the n-back task and backward digit span task compared to other complex verbal span tasks or the forward digit span task.

We propose that the sequencing task shares more similarity with the n-back task in terms of the sequential aspect of information processing. Regular updating occurs as information is presented to the participants [92]. It is interesting to note that only the backward task was specifically associated with a significant increase in response time in the n-back task. In other words, individuals who were more efficient in the backward task took more time to make a decision in the n-back task. They also made significantly fewer commission errors. This correlation could explain the longer response time. Having stronger abilities in the backward task may lead to increased precision in the provided responses. These individuals may be less prone to proactive interference caused by the different words and emotions encountered in the n-back task. They may achieve greater precision by taking more time to resolve conflicts present in memory and make more accurate decisions. In contrast, the overall weak correlation between the two tasks can be explained by the content and different processes involved. On one hand, the digit span task is performed with auditory, neutral, and numerical materials with a verbal response, while the n-back task uses visual, emotional, and textual materials with a motor response. On the other hand, the n-back task may rely on processes of emotional recognition or interference resolution, while the digit span task requires retrieving specific information or manipulating the memorized digits during recall [19,93].

Our study provides new information on the correlations between the digit span tasks, including the rarely studied sequencing task, as well as omission and commission errors in the emotional n-back task. The omission errors did not show any significant correlation with the digit span tasks. However, the commission score exhibited a stronger, significant, negative correlation with all variables of the digit span compared to the accuracy. The strongest correlation was with the total score of the digit span, followed by the Backward task, Sequencing task, and, weakly, with the Forward task and Strategies. We believe that commission scores provide new insights into information processing (the algorithmic level) and decision-making processes (the reflective level). For example, the participants who made fewer commission errors were those who employed more strategies during the tasks. At a minimum, this suggests that individuals seeking optimization of their performance in the digit span task were the ones making fewer commissions in the n-back task. These individuals may be particularly motivated to succeed in the task and pay more attention to what they need to do. This could lead them to respond less systematically and more accurately. However, neither the accuracy score nor the omission score showed a significant correlation with Strategies. Another possible explanation is that the use of strategies may also occur during the n-back task in a search for greater efficiency. This emphasizes the importance of considering the reflective level to understand what these tasks can measure. Although we received qualitative feedback on the implementation of strategies during the n-back task, we did not quantify these observations. Future studies should focus on developing means to verify the observed link. Importantly, while the influence of strategies on the digit span tasks was minimal, the opposite was observed for the n-back task. It is possible that the use of verbal material with meaning may depend more specifically on the reflective level than the use of other types of material. The interference of the reflective level may be more significant in this case. This hypothesis has long guided anxiety theorists in differentiating visual and verbal content, seeking to demonstrate the greater impact of anxiety on verbal material [94]. Furthermore, Redick and Lindsey [91] found higher correlations when complex span and n-back tasks both used visuospatial content and weaker correlations when the content was verbal. We believe that visuospatial tasks are more likely to reflect algorithmic information processing processes, while verbal tasks may be more involved in both levels, especially if emotional content is related to the material.

### 4.3. Attentional Control Theory: Reflexive and Algorithmic Levels

#### 4.3.1. Correlation between Our Variables

Our initial analyses aimed to evaluate the existing links between reflective variables (anxiety, metacognitive beliefs, and ERS) and various working memory measures. Effectiveness was not linked to these variables for either the n-back (accuracy score) or the digit span (standard score). However, the processing efficiency, represented by RTs, omission and commission errors, and the number of strategies used during the digit span, was significantly related to the reflective variables. Trait anxiety, metacognitive beliefs, and maladaptive Emotion Regulation Strategies (ERSms) were positively and significantly associated with RTs.

According to the ACT, we expected that our reflective variables could have more negative effects on the demand and emotional load of a task, due to higher processing costs. The success rate in the n-back task, equivalent to previous experiments [95], supports the difficulty of our task. Although no correlation was found with the accuracy scores, the use of additional indicators, such as the type of error made, was relevant for understanding the involvement of our reflective variables in participants’ decision-making. It is interesting to note that anxiety, ERSms, and metacognitive beliefs provided very similar information regarding omission errors (total omission or emotional alternation omission) and RTs, with a slight difference. Metacognitive beliefs had stronger and more significant correlations, especially for sequences with emotional alternations. Metacognitive beliefs highlight the role of worrying, repetitive, and negative thoughts that can amplify or maintain anxiety. Thoughts such as “I should not worry” might complicate the process of retrieving information in WM and decision-making. This could be particularly true when emotional sequences alternate, creating more confusion and difficulty in decision-making. These thoughts would generate interference in information processing, inducing an overload of working memory (WM). Minor differences in terms of correlation were noted in the links between our anxiety, ERSms, and omission errors for sequences that alternated positive or negative responses. However, omissions were slightly more pronounced for anxiety, as well as for ERSms, on negative response sequences, indicating increased sensitivity to these sequences. A possible explanation for this observation is related to the nature of negative stimuli. Negative emotional stimuli, such as threatening or anxiety-inducing stimuli, can have a stronger impact on the emotional and cognitive state of individuals. In this regard, one study found that anxious individuals showed delays in disengaging from threatening cues, leading to slower responses to non-targets. These delays in disengagement were interpreted as difficulties in diverting attention from cues rather than an initial bias towards the cues themselves [96].

On the other hand, it is interesting to note that only ERSms were negatively correlated with negative commission errors. This might result from increased attention to negative stimuli and reduced accuracy in detecting positive or neutral stimuli. In other words, individuals may be more sensitive, precise, and demanding regarding negative stimuli, leading to fewer commission errors and more omissions. This interpretation is supported by the observed links between ERSms and the number of strategies used in the digit span task. The more individuals use ERSms, the more strategies they employ. We proposed that increased strategy use might reflect significant individual investment in a task, with a desire to succeed and efficiently mobilize their capabilities. These individuals might also be more prone to have negative or worrying thoughts during the digit span task. Further analyses highlighted that negative commission errors and the number of strategies were specifically related to dramatization and Rumination. These ERSms tend to increase the salience of negative stimuli, one through amplification and the other through repetition. This dynamic might explain the negative correlation observed with negative commission errors and the positive correlation with an increased number of strategies. Indeed, these ERSms are associated with unpleasant situations, which might trigger the need to improve one’s performance by generating more strategies. Conversely, Self-blame or Blaming Others is associated with the cause of the unpleasant situation. These strategies would not encourage a proactive approach in seeking solutions. Dramatization and Rumination could be considered mechanisms that prompt the use of costly compensatory strategies in the ACT.

#### 4.3.2. Causal Relationship of Reflective Variables on Working Memory

The ACT proposes that compensatory strategies employed by anxious individuals can lead to additional processing costs (efficiency), which is reflected in behavioral measures such as longer response times without necessarily impacting effectiveness [27]. It suggests that strategies are implemented by the most anxious participants to try to counterbalance their difficulty in accomplishing a task. Our results provide details by highlighting the involvement of metacognitive beliefs and especially ERSms.

To test the influence of our reflective variables on working memory, we conducted several regression analyses. They showed that only ERSms predicted longer RTs and increased uses of strategy in the digit span task. The absence of anxiety’s involvement in these two variables might seem contrary to the ACT, but subsequent mediation analyses allow for another interpretation. Indeed, anxiety did not directly influence the RTs or strategy use, but did so through ERSms. This discovery underscores the importance of reflexive processes engaged during task resolution. It supports the viewpoint of the ACT by foregrounding the reflexive processes of emotion regulation. The cost of regulating emotions maladaptively during a task increases the RT by increasing the implementation of additional strategies. This might be due to individuals using ERSms actively and laboriously seeking ways to manage their emotions or anxiety during the task, which could lead them to try multiple approaches or different strategies. These processes would induce additional processing costs (efficiency) without repercussions on effectiveness. Furthermore, the lack of confidence in one’s cognitive abilities predicting longer response times further reinforces this interpretation.

An increase in the use of ERSms was correlated with an increased frequency of omissions and a decrease in commission errors. This might suggest that participants were adopting a more perfectionist approach in their responses, choosing not to respond (which explains the high number of omissions) to avoid making mistakes (hence the decrease in commissions). However, this interpretation seems limited due to the absence of correlation between the omission and commission scores, as well as the absence of correlation between the response times (RTs) and commission errors. These observations suggest that the underlying mechanisms influencing both omissions and commission errors might be more complex, involving distinct and independent aspects. We suggested that the generation of intrusive thoughts could increase the number of omissions, while an attentional bias towards negative stimuli might decrease the number of commissions with negative emotional alternation. Nevertheless, it is surprising to observe this exclusively for maladaptive ERSs and not for anxiety and metacognitive beliefs, given that these variables are strongly correlated. It is possible that ERSms have a more direct and specific impact on behavior in working memory tasks. These strategies can lead to reflexive processes such as Rumination or dramatization, which interfere more directly with the task than general states of anxiety or metacognitive beliefs. However, the exact nature of these relationships and the underlying mechanisms warrant further exploration in future research.

### 4.4. Perspective of Practical Applications

Several practical applications of our findings can be envisioned. In terms of research, our study highlights the importance of reflexive factors in the ACT and in understanding what working memory tasks measure. The digit span task has been somewhat overlooked in research on working memory assessment, even though it is widely used in neuropsychological and speech therapy assessments. Clinicians are trained to carefully interpret the results of such tests to draw valid conclusions about an individual’s cognitive functioning [17]. However, we have shown that measuring the level of reflexivity can offer a more comprehensive understanding of the mechanisms involved in information processing. Our research on this task is preliminary and could be continued by considering other measures, such as the subject’s awareness/confidence in their own abilities or changes in strategies employed during the task. Our results highlight the need to more broadly incorporate these reflexive processes into the ACT. In this regard, emotion regulation plays an important role, which is logical given its strong connections with executive functions. This experiment, combined with that of Cécillon et al. [26], suggests that conscious processes during a task impact the efficiency of information processing, which can then have significant implications for everyday behaviors requiring executive functions. Working on anxiety, metacognitive beliefs, or emotion regulation could help streamline the use of the algorithmic level and optimize the abilities of adolescents and students for better academic success. For example, paying attention to emotional content in educational tasks seems to be crucial to develop strategies that encourage ERSs. Attentional control theory principles should guide the incorporation of anxiety management strategies and the validation of strategic thinking during learning activities. Differentiated learning materials based on the material type and the promotion of emotional awareness can further contribute to optimizing cognitive processes and academic performance.

### 4.5. Limitations

This study’s stability, aligned with Cécillon et al. [26] and the broader literature, supports its internal validity. Our observations across diverse groups (adolescents, young adults) suggest good external validity. However, the focus on French-speaking, highly educated populations may limit its broader applicability, especially considering the cultural sensitivity of certain processes like ERSs [97].

This study utilized n-back and digit span tasks, differing significantly yet sharing underlying processes, as evidenced by reflective variables such as ERSms. While only two of many methods to assess working memory (WM) were studied, the study’s aim was not to evaluate these tasks’ representation of WM but to show trait anxiety’s impact on them, irrespective of their executive function loading. The n-back task focuses on emotional recognition, and the digit span on simpler memory span tasks. The findings we have obtained on these tasks warrant replication on more common WM tasks.

We can also question the relevance of the tools used. Despite criticisms of assessing unclear concepts [98] and potential overlap with depression [99], STAI is supported by its proven reliability, ability to differentiate anxiety types, and numerous validations [74,100]. Our results are reassuring as they show convergences that are consistent with the literature. For example, the MCneg subscale of the MCQ has been consistently linked to trait anxiety in many studies [49,76,77], specifically to pathological worry [101], a central component of anxiety. Our results largely support these findings and allow us to consider the STAI as a reliable tool for assessing vulnerability to anxiety.

Improvements might involve revised questionnaires following our suggestions and additional tasks for enhanced robustness and broader insights. This preliminary study, examining numerous variables, warrants cautious interpretation. Additionally, we did not clearly distinguish between hot and cold executive functions, as the tasks were significantly different in content and administration modalities.

## 5. Conclusions

This study aimed to (1) understand the mechanisms of trait anxiety and its correlates and (2) assess the influence of the reflective level on working memory (WM) within the theoretical framework of the ACT. Our results highlight the complexity of the interactions between trait anxiety, WM, and decision-making processes related to trait anxiety, metacognitive beliefs, and maladaptive Emotion Regulation Strategies (ERSm). Firstly, the overall variables and their correlations share strong similarities with the study by Cécillon et al. [26], conducted on an adolescent population. Some subscales showed particularly strong correlations with trait anxiety, such as ERSms, while others reliably predicted anxiety, such as MCneg. The relevance of subscales like Blaming Others (CERQ) and Consciousness (MCQ) has been discussed in the context of evaluating anxiety. Secondly, this study highlights the importance of considering both the reflective level (beliefs and strategies) and the algorithmic level (task performance) to understand the underlying mechanisms of cognitive performance in the context of the ACT. Metacognitive beliefs as well as ERSms were differently associated with the types of errors in the n-back task and the number of strategies used during the digit span task. These pieces of information were non-redundant and provided new considerations in comparison to trait anxiety. Moreover, considering these reflective variables demonstrated that anxiety had an indirect effect on the algorithmic level through ERSms. These predicted a higher processing cost with increased RTs and a greater number of strategies used in the digit span task. This study further elaborates on the ACT by highlighting the importance of reflexive processes, including emotion regulation, in the processing cost of information in WM.

## Figures and Tables

**Table 1:** Descriptive statistics of Working Memory tasks.

Working Memory Tasks		*n* (Missing)	Mean	SD	Min	Max
Digit Span	Total score	110	9.518	2.566	4.00	17.00
	Strategies	109 (1)	2.358	0.866	1.00	5.00
n-back	Accuracy	110	0.65	0.119	0.375	0.966
	Response Time Total	110	1423.50	292.52	705.98	1999.32
	Omission	110	5.661	7.755	0.00	39.773
	Commission	110	0.283	0.090	0.034	0.50
	Positive Omission Errors	110	0.058	0.091	0.00	0.50
	Negative Omission Errors	110	0.048	0.083	0.00	0.417
	Positive Commission Errors	110	0.271	0.150	0.00	0.667
	Negative Commission Errors	110	0.375	0.194	0.00	1.00
	Emotional Total Omission	110	0.053	0.078	0.00	0.375
	Emotional Total Commission	110	0.323	0.134	0.00	0.708

Conditioned on variable: sex. Strategies = number of strategies used during the Digit Span Task.

**Table 2:** Pearson correlations between working memory tasks.

Variables		n-Back				Digit Span			
		Accuracy	RT	Omission	Com	Total Score	Strategies	Forward	Backward
n-back	RT	−0.074	—						
	Omission	−0.614 **	0.295 **	—					
	Commission	−0.736 **	−0.159	−0.067	—				
Digit span	Total score	0.358 **	0.156	−0.036	−0.445 **	—			
	Strategies	0.127	0.033	0.084	−0.224 *	0.109	—		
	Forward	0.215 *	0.028	−0.094	−0.213 *	0.706 **	0.109	—	
	Backward	0.261 **	0.192 *	0.050	−0.389 **	0.753 **	0.079	0.377 **	—
	Sequencing	0.279 **	0.124	−0.036	−0.329 **	0.705 **	0.078	0.233 *	0.301 **

Conditioned on variable: sex. RT = Response Time; Com = Commission; Strategies = Number of strategies used during the digit span task. * *p* < 0.05 ** *p* ≤ 0.006.

**Table 3:** Pearson correlations between main variables.

Variables			ERS		
		MCQ	Maladaptive	Adaptive	STAI
ERS	Maladaptive	0.519 **	—		
	Adaptive	0.038	−0.066	—	
n-back	STAI	0.592 **	0.569 **	−0.169	—
	Accuracy	−0.103	0.050	0.064	−0.096
	Response Time	0.195 *	0.308 **	−0.120	0.205 *
	Commission	−0.040	−0.214 *	0.058	−0.017
	Emotional Total Commission	−0.068	−0.151	0.004	−0.005
	Positive Commission Errors	−0.050	0.038	0.049	−0.035
	Negative Commission Errors	−0.056	−0.238 *	−0.032	0.021
	Omission	0.200 *	0.181	−0.187	0.187
	Emotional Total Omission	0.296 **	0.191 *	−0.138	0.178
	Positive Omission Errors	0.266 **	0.154	−0.126	0.127
	Negative Omission Errors	0.266 **	0.193 *	−0.122	0.197 *
Digit span	Total score	−0.103	0.040	0.004	−0.034
	Strategies	−0.000	0.263 **	0.012	0.014

Conditioned on variable: sex. ERS = Emotion Regulation Strategies; * *p* < 0.05 ** *p* ≤ 0.006.

## Data Availability

CECILLON, François-Xavier (2024), “Study 2023”, Mendeley Data, V3, accessed on 23 February 2024. https://data.mendeley.com/datasets/z4fdg43ynn/3.

## Supplementary Materials

The following supporting information can be downloaded at: https://www.mdpi.com/article/10.3390/ejihpe14030034/s1 and CECILLON, François-Xavier (2024), “Study 2023”, Mendeley Data, V3, accessed on 23 February 2024. https://data.mendeley.com/datasets/z4fdg43ynn/3. Found in the supplementary material: A more detailed description of the n-back task equipment, Additional analyses on gender differences in our sample, and the analyses of the curvilinear relationship between anxiety and our variables, as all analyses indicate a linear relationship. References [102,103,104,105,106,107,108,109,110,111,112,113,114,115,116,117,118,119] are also included in Supplementary Materials.
