# Supplementary material for: The Reflective Mind of the Anxious in Action: Metacognitive Beliefs and Maladaptive Emotional Regulation Strategies Constrain Working Memory Efficiency
Source: Eur J Investig Health Psychol Educ. 2024 Feb 26;14(3):505–30. doi: 10.3390/ejihpe14030034 (PMC10968987; doi:10.3390/ejihpe14030034)
Supplement: Supplementary file 1 [file ejihpe-14-00034-s001.zip › ejihpe-2795837-supplementary.pdf]

Supplementary Materials

This supplementary material includes statistical analyses regarding (1) differences between men and women across all our variables and (2) the analysis of anxiety effects across three intensity levels (low, moderate, high) on our variables of interest. These analyses mirror those conducted in Cécillon et al. (2023). The aim is to discuss the replicability of these results within a different population.

3. Results

For independent group comparisons, a Welch's analysis was applied by default, following the recommendation of Delacre, Lakens, and Leys [1].

3.1. Sex differences

We conducted a Welch's t-test to determine if there were differences in anxiety levels between male and female participants. Our results indicate that girls reported significantly higher levels of anxiety (M = 52.47, SD = 9.97) compared to boys (M = 44.78, SD = 9.08) (t = 4.036, p < .001).

Table 1. Welch's t-test between boys and girls on emotion regulation strategies.

Variables	t	df
Maladaptive emotion regulation strategies	.574	60.014
Adaptive emotion regulation strategies	-2.171*	73.605
Acceptance	-.299	66.991
Positive refocusing	-1.529	52.204
Refocus on planning	-1.625	88.440
Positive reappraisal	-3.402**	73.176
Putting into perspective	-.146	67.551
Self-blame	.988	69.158
Rumination	.739	64.491
Catastrophizing	-.045	57.032
Blaming others	-.619	70.336

* p = .033 ** p < .01

We conducted a Welch's t-test to determine if emotion regulation strategies and metacognitions were dependent on participants' gender (Table 1).

The results did not indicate any differences in the use of maladaptive strategies between boys and girls, but there was a slight difference in the use of reappraisal (Mean difference = 5.59, SD = 13.22 for girls and SD = 12.40 for boys; p = .033). In terms of SRE, boys significantly used more reappraisal (M = 15.28, SD = 3.84) than girls (M = 12.57, SD = 4.07) with p = .001.

Regarding the MCQ, there were no significant differences in the total scale. However, we observed a significant difference in the negative beliefs subscale (t = 2.64, df = 78.96, p = .010). Girls had stronger negative beliefs than boys (respectively, M = 16.49, SD = 4.28 and M = 14.39, SD = 3.71). In accordance with Esbjorn et al. [2], we conducted an ANCOVA, controlling for participants' anxiety, using JASP. The results indicated that anxiety nullifies the observed difference for negative metacognitive beliefs: $F_{1,109}$ = .238, p = .627.

We also tested our sample on the working memory indices of our study. The only significant differences found were in the total standard score of the digit span (t = -2.247, p = .028) and in the total reaction time of the n-back task (t = -2.012, p = .048). Men displayed better performance in the digit span, which was explained by a significant difference in the standard score for sequencing (t = -2.780, p = .007). Men were slower than women in the emotional n-back task. There were no differences in the number of strategies used during the digit span task or in the accuracy of the n-back task.

3.2. Curvilinear relationship of anxiety

Prior to conducting our correlation and regression analyses, we examined anxiety based on its intensity: low, moderate, and high. We used the "Percentile" function in Excel and assigned the values 1/3 and 2/3 to obtain the median values of our sample on the STAI (respectively, 43 and 54). The sample with a score equal to or below 43, indicating mild anxiety, consisted of 36 participants, the sample with scores between 44 and 53, indicating moderate anxiety, included 37 participants, and the sample with a score equal to or above 54, indicating high anxiety, included 37 participants. Several ANOVAs (i.e. for each dependent variable) were conducted using JASP to examine the relationship between anxiety intensity and the main variables. Given the significant differences previously identified, sex was consistently entered as a covariable.

Regarding emotion regulation strategies (ERSs), there was a main effect of anxiety on maladaptive ERSs ($F_{2,104}$ = 19.549; p < .001) and adaptive ERSs ($F_{2,104}$ = 4.970; p = .009). According to Tukey's post hoc test, maladaptive ERSs showed a positive and almost linear relationship with anxiety for both boys and girls. In other words, higher anxiety was associated with more maladaptive ERSs. Similarly, adaptive ERSs showed a negative relationship with anxiety. However, for both maladaptive and adaptive ERSs, the differences between mild and moderate anxiety were not significant.

Regarding metacognitions, anxiety had a significant impact on metacognitions ($F_{2,104}$ = 15.068; p < .001). According to Tukey's post hoc test, the main effect of this ANOVA revealed that, as anxiety increased, metacognitions became more pronounced, with no significant difference between moderate and severe anxiety.

There was no effect of sex on metacognitions or on the interaction between gender and anxiety intensity.

Regarding working memory, the ANOVA revealed a main effect of sex ($F_{1,104}$ = 3.929; p < .050) on the total score of the digit span. Boys had a higher score than girls (Mean difference = -1.153; SE = .582; t = -1.982). However, there were no significant differences in the accuracy score, commission score, or omission score of the n-back task, or the number of strategies used during the digit span. In contrast, for response times, the ANOVA revealed two main effects, both for sex ($F_{1,104}$ = 8.664; p = .004) and anxiety intensity ($F_{2,104}$ = 3.656; p = .029). Men were slower than girls in their response times (Mean difference = -190.380; SE = 64.679; t = -2.943). Participants with mild anxiety were significantly faster than those with severe anxiety (Mean difference = -224.389; SE = 83.196; t = -2.697). No interaction effect was observed for our variables related to executive functions.

4. Discussion

The present study aimed to verify and generalize the findings from Cécillon et al. [3] and the literature regarding metacognitive beliefs, ERSs, and trait anxiety. Consistent with previous studies using similar measures, women reported higher levels of trait anxiety compared to men [82–84]. However, it is important to note that these differences are not consistently found across all studies using the STAI [85–87]. A review by McLean and Anderson identified several differences that could explain this discrepancy, including variations in coping strategies [10]. Women tend to use emotion-focused strategies (such as Rumination), while men tend to use problem-solving strategies. Our results showed higher use of a positive reappraisal strategy among men compared to women, but no difference in Rumination. McRae et al. conducted an fMRI study on emotion regulation and suggested that men were likely to use reappraisal more easily than women [11]. Men exhibited more effective regulation of amygdala activity, which is involved in emotional reactivity, and showed less prefrontal cortex activity, which is involved in reappraisal. Considering the absence of differences in negative affect levels and the effective use of reappraisal (similar amygdala activation during emotion and regulation), the authors also proposed that women are more likely to generate a positive affect to regulate negative emotions (based on greater activation of the ventral striatum in women). However, in the CERQ, positive reappraisal has a positive connotation, which should have balanced the difference or, at least, made it weaker. A simpler explanation for our data in terms of ERSs is to consider the differences in the sample proportions between genders.

Regarding the MCQ, there was a difference between women and men on the "negative metacognitive beliefs" subscale (MCneg). However, as with Cécillon et al. [3], this difference

disappeared when controlling for trait anxiety in an ANCOVA. This process has been employed by Esbjørn et al. [2] and supports the strong link between trait anxiety and metacognitive beliefs, including MCneg. The systematic review by Myers et al. [12] identified very small differences in children and adolescents. It appears that enough data have been presented on this topic to conclude, with relatively low risk, that metacognitive beliefs are not likely to explain higher anxiety in women.

We found differences between men and women on our variables of interest for working memory. Men performed better on the digit span task, mainly due to higher scores in the forward and slightly in the backward order processing. Piccardi et al. [13] attempted to observe differences among young adults on digit span tasks (forward and backward) but found none. According to these authors, if differences are found on these tasks, they would be primarily due to sample heterogeneity rather than gender differences, which aligns with our data. However, the meta-analysis by Lynn and Irwing [14] found that gender differences are more likely to be present in samples with lower levels of education. Therefore, it is contradictory to find differences in our university sample. Thus, we consider these results as characteristics of our sample that do not allow for broader conclusions about these processes. We also found differences in response times for the n-back task, with women being faster than men. Contradictory results have been reported on n-back tasks with verbal material, as some studies showed that men were faster than women [92,93], while others found no difference [94,95]. The tasks in the cited studies involved n-back tasks with letter sequences, making them difficult to compare with ours. However, the heterogeneity of results on similar tasks suggests that this type of result may depend on factors independent of the task itself, such as sample characteristics or real differences in cognitive processes between men and women. For example, previous studies have shown that women have advantages in certain tasks measuring working memory, such as verbal fluency tests. In phonemic fluency tests, where participants are asked to generate as many words as possible starting with a specific letter in 60 seconds, women adopt a balanced strategy of word clustering and category switching. In contrast, men tend to change categories less frequently and cluster words more, resulting in a lower total number of generated words [19]. Additionally, other research has shown superior performance by women on the California Verbal Learning Test (CVLT), which assesses various components of verbal memory. This suggests an advantage for women in memorizing verbal material [20]. Women may benefit from the spontaneous use of semantic switching strategies and their advantages in verbal memory in the emotional n-back task. These strategies could facilitate switching from one word to another from a different category, which could improve response times without affecting response accuracy. It is important to note that the task we used is novel, and significant variations have been observed in more established tasks. Therefore, it would be necessary to further evaluate this task to better understand what it measures and obtain clearer results.

References

1. Delacre, M.; Lakens, D.; Leys, C. Why Psychologists Should by Default Use Welch's t-Test Instead of Student's t-Test. *Int. Rev. Soc. Psychol.* **2017**, *30*, 92–101, doi:10.5334/irsp.82.
2. Esbjørn, B.H.; Sømhovd, M.J.; Holm, J.M.; Lønfeldt, N.N.; Bender, P.K.; Nielsen, S.K.; Reinholdt-Dunne, M.L. A Structural Assessment of the 30-Item Metacognitions Questionnaire for Children and Its Relations to Anxiety Symptoms. *Psychol. Assess.* **2013**, *25*, 1211.
3. Cécillon, F.-X.; Mermillod, M.; Lachaux, J.-P.; Shankland, R. Trait Anxiety, Emotion Regulation Strategies, and Metacogitive Beliefs: Their Influence on Executive Functions and Academic Achievement. **2023**, doi:10.17605/OSF.IO/2HXJQ.
4. Alvarez-Vargas, D.; Abad, C.; Pruden, S.M. Spatial Anxiety Mediates the Sex Difference in Adult Mental Rotation Test Performance. *Cogn. Res. Princ. Implic.* **2020**, *5*, 31, doi:10.1186/s41235-020-00231-8.
5. Bander, R.S.; Betz, N.E. The Relationship of Sex and Sex Role to Trait and Situationally Specific Anxiety Types. *J. Res. Personal.* **1981**, *15*, 312–322.
6. Kelly, M.M.; Tyrka, A.R.; Price, L.H.; Carpenter, L.L. Sex Differences in the Use of Coping Strategies: Predictors of Anxiety and Depressive Symptoms. *Depress. Anxiety* **2008**, *25*, 839–846, doi:10.1002/da.20341.

7. Montagne, B.; Kessels, R.P.C.; Frigerio, E.; de Haan, E.H.F.; Perrett, D.I. Sex Differences in the Perception of Affective Facial Expressions: Do Men Really Lack Emotional Sensitivity? *Cogn. Process.* **2005**, *6*, 136–141, doi:10.1007/s10339-005-0050-6.
8. Strand, N.; Fang, L.; Carlson, J.M. Sex Differences in Anxiety: An Investigation of the Moderating Role of Sex in Performance Monitoring and Attentional Bias to Threat in High Trait Anxious Individuals. *Front. Hum. Neurosci.* **2021**, *15*.
9. Zhang, H.; Bi, Y.; Hou, X.; Lu, X.; Tu, Y.; Hu, L. The Role of Negative Emotions in Sex Differences in Pain Sensitivity. *NeuroImage* **2021**, *245*, 118685, doi:10.1016/j.neuroimage.2021.118685.
10. McLean, C.P.; Anderson, E.R. Brave Men and Timid Women? A Review of the Gender Differences in Fear and Anxiety. *Clin. Psychol. Rev.* **2009**, *29*, 496–505, doi:10.1016/j.cpr.2009.05.003.
11. McRae, K.; Ochsner, K.N.; Mauss, I.B.; Gabrieli, J.J.D.; Gross, J.J. Gender Differences in Emotion Regulation: An fMRI Study of Cognitive Reappraisal. *Group Process. Intergroup Relat.* **2008**, *11*, 143–162, doi:10.1177/1368430207088035.
12. Myers, S.G.; Solem, S.; Wells, A. The Metacognitions Questionnaire and Its Derivatives in Children and Adolescents: A Systematic Review of Psychometric Properties. *Front. Psychol.* **2019**, *10*, 1871.
13. Piccardi, L.; D'Antuono, G.; Marin, D.; Boccia, M.; Ciurli, P.; Incoccia, C.; Antonucci, G.; Verde, P.; Guariglia, C. New Evidence for Gender Differences in Performing the Corsi Test but Not the Digit Span: Data from 208 Individuals. *Psychol. Stud.* **2019**, *64*, 411–419.
14. Lynn, R.; Irwing, P. Sex Differences in Mental Arithmetic, Digit Span, and g Defined as Working Memory Capacity. *Intelligence* **2008**, *36*, 226–235, doi:10.1016/j.intell.2007.06.002.
15. Pelegrina, S.; Lechuga, M.T.; García-Madruga, J.A.; Elosúa, M.R.; Macizo, P.; Carreiras, M.; Fuentes, L.J.; Bajo, M.T. Normative Data on the N-Back Task for Children and Young Adolescents. *Front. Psychol.* **2015**, *6*.
16. Speck, O.; Ernst, T.; Braun, J.; Koch, C.; Miller, E.; Chang, L. Gender Differences in the Functional Organization of the Brain for Working Memory. *NeuroReport* **2000**, *11*, 2581.
17. Lejbak, L.; Crossley, M.; Vrbancic, M. A Male Advantage for Spatial and Object but Not Verbal Working Memory Using the N-Back Task. *Brain Cogn.* **2011**, *76*, 191–196, doi:10.1016/j.bandc.2010.12.002.
18. Schmidt, H.; Jogia, J.; Fast, K.; Christodoulou, T.; Haldane, M.; Kumari, V.; Frangou, S. No Gender Differences in Brain Activation during the N-Back Task: An fMRI Study in Healthy Individuals. *Hum. Brain Mapp.* **2009**, *30*, 3609–3615, doi:10.1002/hbm.20783.
19. Weiss, E.M.; Ragland, J.D.; Brensinger, C.M.; Bilker, W.B.; Deisenhammer, E.A.; Delazer, M. Sex Differences in Clustering and Switching in Verbal Fluency Tasks. *J. Int. Neuropsychol. Soc.* **2006**, *12*, 502–509, doi:10.1017/S1355617706060656.
20. Norman, M.A.; Evans, J.D.; Miller, W.S.; Heaton, R.K. Demographically Corrected Norms for the California Verbal Learning Test. *J. Clin. Exp. Neuropsychol.* **2000**, *22*, 80–94, doi:10.1076/1380-3395(200002)22:1;1-8;FT080.
